# Awareness and practices regarding antimicrobial resistance among livestock farmers in Northern Uganda

**DOI:** 10.3389/frabi.2026.1745965

**Published:** 2026-04-06

**Authors:** John Dickens Kato, Peace Okello Lamaro, John Paul Waiswa, Grace Madraa, Donald Otika, Morrish Obol Okello, Daniel S. Ebbs, Pebalo Francis Pebolo, Felix Bongomin

**Affiliations:** 1 Faculty of Medicine, Gulu University, Gulu, Uganda; 2 Faculty of Agriculture, Gulu University, Gulu, Uganda; 3 Pediatric Critical Care Medicine, Yale University School of Medicine, New Haven, CT, United States

**Keywords:** antimicrobial resistance, antimicrobial use, antimicrobials, livestock farming, Northern Uganda

## Abstract

**Background:**

Antimicrobial use in animals may contribute to antimicrobial resistance (AMR), which poses direct risks to animal health and welfare and can potentially impact human health since some diseases affect both animals and people. This study determined the level of awareness regarding AMR among livestock farmers in Northern Uganda.

**Methods:**

A community-based cross-sectional study was conducted among livestock farmers in three districts of Northern Uganda, namely, Gulu, Omoro, and Amuru districts. A structured questionnaire was used to collect data.

**Results:**

Data from 246 participants, with a median age of 38 years (interquartile range: 29–50 years), were analyzed. Most farmers had attained a primary level of education (*n* = 125, 50.8%) and grew crops as their major economic activity (*n* = 88, 35.8%). Goats were the most commonly reared animals (*n* = 167, 67.7%). The median distance from the nearest drug shop was 2 km (interquartile range: 1–5 km). Most farmers had good awareness on AMR (158, 64.2%), with more than half of the farmers (*n* = 134, 54.5%) having heard of AMR, but only 48 (35.8%) understood its correct meaning. Most farmers (*n* = 220, 89.4%) noted AMR as an important issue in farming, with many farmers (*n* = 133, 54.5%) opting to seek veterinary advice for prevention. Most farmers had appropriate AMU (219, 89.0%), with 203 (82.5%) having used antimicrobials in the last 12 months. Most farmers (*n* = 184, 74.8%) obtained drugs and dosage from veterinary doctors, with majority following the recommended dosage (*n* = 227, 92.3%) and proper withdrawal periods (*n* = 221, 89.8%). Overall, few farmers (*n* = 81, 32.9%) received training on AMR, with more than half getting training from veterinary professionals (*n* = 41, 55.4%). The major source of information was veterinary professionals (*n* = 181, 73.6%), followed by radio stations (*n* = 160, 65.0%). Using logistic regression where *P* < 0.05 was considered statistically significant, secondary education was the only factor significantly influencing AMR awareness at multivariable analysis (aOR: 1.85, 95% CI: 0.80–4.26, *P* = 0.030), while age group 52–85 years was the only factor significantly associated with appropriate practices at both bivariate analysis (cOR: 8.10, 95% CI: 1.07–61.37, *P* = 0.043) and multivariable analysis with a stronger significance (aOR: 11.19, 95% CI: 1.50–83.36, *P* = 0.018). A chi-square analysis was done where *P* < 0.05 was considered statistically significant, and it showed that there was a significant difference to access to veterinary services across districts (*P* = 0.014) and a highly significant association between training and appropriate practices in farmers (*X*^2^ (1) = 13.048, *P* = 0.000).

**Conclusion:**

Most livestock farmers had limited understanding of what AMR precisely means, which highlights a significant knowledge gap.

## Introduction

Livestock such as goats and cattle are widely produced around the world, primarily for food consumption and financial gain (Ilea, 2009). Livestock farming thus refers to raising and managing animals for food, fiber, and other products. However, there are challenges associated with livestock farming, particularly regarding AMR (Ayukekbong et al., 2017).

Antimicrobials including antibiotics, antivirals, antifungals, and antiparasitic are medicines used to prevent and treat infectious diseases in humans, animals, and plants (Puvača, 2022). Antimicrobial resistance (AMR) occurs when bacteria, viruses, fungi, and parasites change over time and no longer respond to antimicrobials to which they were previously susceptible (Getahun, 2023). AMR has emerged as one of the principal public health problems of the 21st century that threatens the effective prevention and treatment of an ever-increasing range of infections that are no longer susceptible to the medicines commonly used to treat them (Prestinaci et al., 2015).

In 2021, an estimated 4.71 million deaths were associated with bacterial AMR, including 1.14 million deaths attributable to bacterial AMR aureus which increased the most. Among Gram-negative bacteria, resistance to carbapenems increased more than any other antibiotic class (Naghavi et al., 2024). Resistance is spread through both vertical gene transfer (parent to offspring) as well as by horizontal gene transfer like transformation, transduction, and conjugation (Nadeem et al., 2020). Water, soil, and wildlife represent important AMR reservoirs. Thus, AMR is one of the most important human- and animal-health-threatening issues worldwide (Palma et al., 2020).

The misuse and overuse of antimicrobials are the main drivers in the development of drug-resistant pathogens often unnecessarily or without a prescription (Getahun, 2023). The use of antimicrobials in food-producing animals has increased in the past decades due to a rise in the global demand for animal protein (Nadeem et al., 2020). Antibiotics are also used as growth promoters, which involves administering low doses (sub-therapeutic) of antibiotics in feed or water over an extended period of time to improve growth and production efficiency in countries such as India and China (Canton et al., 2021).

In developing countries, there is evidence on most aspects of agricultural-related AMR which includes the use of antimicrobials in agriculture, impacts of this use on human and animal health, the acceptability and feasibility of stricter control of antibiotic use in agriculture, and the costs and benefits of stricter control, taking into account trade-offs between overuse and lack of access to antimicrobial drugs (Grace, 2015).

The most frequently used classes of antibiotics in animals are quinolones (especially fluoroquinolones), aminopenicillins alone, or in combination with potentiators, first- and second-generation cephalosporins, tetracyclines, sulfonamides alone, or in combination with potentiators, cephalosporins, macrolides, and glycopeptides (Broens and van Geijlswijk, 2018).

In a study done in Mymensingh Division of Bangladesh, 71.2% of the farmers had heard about antimicrobial resistance (Hossain et al., 2022), with studies done in different African countries which revealed that 83% of livestock farmers get antibiotics from agro-veterinary pharmacies (Caudell et al., 2020), while 45.5% of northeastern Ethiopia farmers get antimicrobials from veterinary pharmacies (Gebeyehu et al., 2021).

In Kenya, a study showed that 85.7% had heard of AMR before (Omolo et al., 2024). In Kiambu County, Kenya, 78% reported the use of an antibiotic in the past 6 months prior to the study (Kariuki et al., 2023). Another study showed that almost all farms (92.7%) reported administering antibiotics to their cattle at least once in the last year (Kisoo et al., 2023).

In a study done in Wakiso District, Uganda, 63.6% had heard about AMR, with 37.1% participants mentioning that AMR was only a problem for people who took antimicrobials regularly, but almost all of the participants (96.7%) agreed that people should use antimicrobials only when prescribed by a health professional. The farmers also obtained antimicrobials for use in animals from veterinary workers (74.5%) and veterinary drug shops (19.1%) (Musoke et al., 2023).

Therefore, information regarding antimicrobial use and AMR in a specific area is crucial, and there is a need to identify farmers’ risky behavior and factors associated with them as possible targets for intervention (Caudell et al., 2020). There is paucity of data on KAP about AMR among livestock in northern Uganda, especially in Omoro, Gulu, and Amuru districts, though these areas heavily rely on livestock as a source of income to support their home. This study is therefore aimed at assessing the awareness and practices regarding AMR among livestock farmers in Omoro, Gulu, and Amuru districts in Northern Uganda.

## Methods

### Study design

We conducted a community-based cross-sectional study employing a quantitative approach to assess the awareness and practices regarding AMR among livestock farmers in Omoro, Gulu, and Amuru districts of Northern Uganda.

### Study setting

This study was conducted from three districts of the Acholi subregion of northern Uganda: Gulu—2.768° N, 32.296° E; Omoro—2.708° N, 32.307° E; and Amuru—3.000° N, 32.891° E. These districts in northern Uganda have agriculture-based economies with livestock farming being a major source of livelihood, with the farmers rearing animals such as cattle mostly and also others such as sheep and goats for milk, meat, and breeding purposes. There are government veterinary offices in all of these districts, with a few staff and limited services. Thus, most farmers use many private shops. Most farmers access information about AMR from fellow farmers and radio stations.

### Study population

Small- and large-scale livestock farmers in Omoro, Gulu, and Amuru districts of Northern Uganda who were above 18 years of age and have farmed for more than 3 years comprised the study population.

### Sample size

We used Cochran’s formula:


$$n_{0}=\frac{Z^{2}pq}{e^{2}}$$

where:

*n*_0_ = sample size.
*Z* = *Z*-score corresponding to the desired confidence level (1.96 for 95% confidence level).
*p* = estimated population proportion using *P* = 0.8, estimated number of livestock farmers in Uganda (Survey & Release, 2022).
*q* = 1 – *p.*
*e* = margin of error (set as 0.05).
*n*_0_ = (1.96^2^ × 0.8 × (1 - 0.8))/0.05^2^ = 246 participants.

### Sampling method

The stratified sampling method was based on the geographic distribution within the three districts selected. The strata contained two divisions/parishes, and within each sub-stratum, villages were chosen. Then, random sampling was conducted, where the interviewers moved with local council leaders, from one homestead to the next, in visiting any of the livestock farmers present in the village who met the criteria. In Gulu, the study was conducted in two villages of Paicho subcounty (Wigweng and Ajanyi) and two villages in Bungatira subcounty, namely, Ayac and Layik West. In Amuru district, the study was conducted in Amuru Town Council in Pogi and Lukungu villages and Palema division in Amilobo and Luyaguma villages. In Omoro district, the study was done in Koro subcounty: Palami and Tyen Akaya B villages and in Omoro Town Council in Arwotomiya and Okar villages.

### Data collection

Data were collected through structured interviews administered to eligible study participants using a structured questionnaire. Trained interviewers fluent in both English and Acholi administered the interviews face-to-face. The interviews were conducted in a safe and private space to maintain ethical guidelines. Data from the interviews were thereafter entered into a secure electronic database.

A total of 250 eligible participants were approached, where 246 consented and were interviewed for the study—thus giving a response rate of 98.4%.

### Data analysis

Data were analyzed using STATA 15. To determine the level of awareness, attitude, and practices about AMR among livestock farmers in Omoro, Gulu, and Amuru districts in Northern Uganda, we used descriptive statistics, categorical variables were summarized using frequency and percentage, and numerical variables were summarized using median and inter-quartile range as the data were not normally distributed.

### Ethical considerations

Ethical approval was obtained from the Gulu University Research Ethics as GUREC-2024-1066. Administrative clearances were thereafter obtained from the Chief Administrative Officer and the district veterinary officer of the selected study sites. Access to the local farmers was requested from the area leaders. Informed consent was obtained from all participants, to whom the study’s purpose and benefits were clearly outlined. Participant confidentiality and anonymity were also maintained throughout the study, and the participants had the right to withdraw from taking part in the study at any point without any repercussions. The study was conducted according to the Declaration of Helsinki regarding autonomy, beneficence, non-maleficence, and justice of participants.

## Results

### Socio-demographic characteristics of livestock farmers

Out of the 246 participants interviewed, the median age was 38 years (interquartile range: 29–50 years). Most farmers had primary education (125, 50.8%) and grew crops as their major economic activity (88, 35.8%). Goats were the most commonly reared animals (167, 67.7%), with a median of 10 animals (interquartile range: 6–19). The median distance from the nearest drug shop was 2 km (interquartile range: 1–5), and few farmers (15, 6.1%) kept exotic breeds. The median estimated monthly income was 100,000 Ugandan shillings (interquartile range: 30,000–200,000) (**Table 1**).

**Table 1:** Socio-demographic characteristics of the livestock farmers.

Variables	Frequency	Percentage	Median, (interquartile range)
Gender			
Male	124	50.4	
Female	122	49.6	
Age (median (interquartile range))			38 (29–50)
District			
Amuru	82	33.3	
Gulu	82	33.3	
Omoro	82	33.3	
Level of education			
No formal education	39	15.9	
Primary education	125	50.8	
Secondary education	60	24.4	
Tertiary education	22	9.0	
Type of animal			
Cattle	123	50	
Pigs	78	31.7	
Goats	167	67.9	
Sheep	9	3.7	
Rabbits	10	4.1	
Chickens	94	38.2	
Number of animals (median (interquartile range))			10 (6–19)
Distance from drug shop (median (interquartile range))	2	1–5	
Estimated monthly income (median (interquartile range))			100,000 (30,000–200,000)
Main economic activity			
Livestock keeping	49	19.92	
Crop growing	88	35.8	
Business	33	13.4	
Both livestock and crop farming	76	30.9	
Number of farmers who keep exotic breeds	15	6.1	

### Awareness on antimicrobial resistance among livestock farmers

Most farmers had good awareness on AMR (158, 64.2%), with more than half of the farmers (134, 54.5%) having heard of AMR; however, only 48 (35.8%) understood its correct meaning. Majority of them (220, 89.4%) noted antimicrobial resistance as important in farming. Most farmers (86, 35.0%) stated that antimicrobials work by preventing disease progression. The main causes of AMR selected were lack of proper vaccination (89, 36.5%) and overuse of antibiotics (88, 36.1%). Most farmers reported increased treatment costs (123, 50.4%) as the main effect of antimicrobial resistance, and more than half stated that it can be prevented by seeking veterinary advice (133, 54.5%) (**Table 2**).

**Table 2:** Awareness about antimicrobial resistance among livestock farmers.

Variables	Sample size	Frequency (percentage)
AMR awareness	246	
Good		158 (64.2%)
Poor		88 (35.8%)
Heard of antimicrobial resistance	246	134 (54.5%)
Know the meaning of antimicrobial resistance	134	48 (35.8%)
How antimicrobials work	246	
Kill the organisms causing the disease		78 (31.7%)
Prevent the disease from progressing		86 (35.0%)
Boost the immunity of the animal		82 (33.3%)
Importance of antimicrobial resistance in farming	246	
Not important		26 (10.6%)
Important		220 (89.4%)
Causes of antimicrobial resistance	243	
Overuse of antibiotics		88 (36.1%)
Poor hygiene		85 (34.8%)
Contaminated feed		73 (29.9%)
Lack of proper vaccination		89 (36.5%)
Effects of antimicrobial resistance	245	
Reduced animal health		117 (48.0%)
Increased treatment costs		123 (50.4%)
Decreased productivity		61 (25.0%)
Higher risk of disease spread		87 (35.7%)
Prevention of antimicrobial resistance	246	
Improved farm hygiene		85 (34.8%)
Rational antibiotic		84 (34.4%)
Regular vaccination		91 (37.3%)
Avoiding contamination		58 (23.8%)
Seeking veterinary advice		133 (54.5%)

### Practices about antimicrobial resistance among livestock farmers

Most farmers had appropriate AMU (219, 89.0%), with 203 (82.5%) having used antimicrobials in the last 12 months. Veterinary prescription guided the antimicrobial choice selection (180, 73.2%), where most farmers obtained the drugs and dosage from veterinary doctors (184, 74.8%). Most of the farmers used antibiotics rarely (158, 64.2), with majority following the recommended dosage (227, 92.3%) and proper withdrawal periods (221, 89.8%). Overall, 81 (32.9%) received training on antimicrobial resistance, with more than half getting training from veterinary professionals (41, 55.4%) (**Table 3**).

**Table 3:** Practices about antimicrobial resistance among livestock farmers.

Variables	Sample size	Frequency	Percentage
Practices	246		
Appropriate		219	89.0
Inappropriate		27	11.0
Use of antimicrobial in last 12 months	246	203	82.5
Frequency of antibiotics use	246		
Weekly		12	4.9
Monthly		76	30.9
Rarely		158	64.2
Obtaining antibiotics and their dosage	246		
Veterinary doctors		184	74.8
Drug shops		48	19.5
Fellow farmers		2	0.8
Personal experience		5	2.0
Drug bottle		7	2.9
Following recommended dosage	246	227	92.3
Following proper withdrawal periods for antibiotics	246	221	89.8
Training on antimicrobial resistance	246	81	32.9
Source of training	74		
Veterinary professional		41	55.4
Private organization		17	23.0
Government health organization		4	5.4
NGO		11	14.9
Other		1	1.3
Determination of antimicrobial choice	246		
Veterinary prescription		180	73.2
Personal experience		28	11.4
Cost		13	5.3
Recommendations from other farmers		25	10.2

### Bivariate and multivariable analysis of factors influencing AMR awareness

In the bivariate analysis using simple logistic regression, where *P* < 0.05 was considered statistically significant, no factors were found to be significant. In the multivariable analysis using logistic regression where *P* < 0.05 was considered statistically significant; secondary education was the only significant factor (aOR: 1.85, 95% CI: 0.80–4.26, *P* = 0.030) (**Table 4**).

**Table 4:** Bivariate and multivariable analysis of factors influencing AMR awareness.

Variables	Good AMR awareness	Poor AMR awareness	cOR (95% CI)	*P*-value	aOR (95% CI)	*P*-value
Age						
18–51	120 (62.18%)	73 (37.82%)	(ref)		(ref)	
52–85	38 (71.70%)	15 (28.30%)	1.54 (0.79–3.00)	0.203	1.89 (0.91–2.91)	0.083
Gender						
Male	79 (63.71%)	45 (36.29%)	(ref)		(ref)	
Female	79 (64.75%)	43 (35.25%)	1.05 (0.62–1.76)	0.865	1.33 (0.75–2.35)	0.329
District						
Amuru	46 (56.10%)	36 (43.90%)	(ref)		(ref)	
Gulu	56 (68.29%)	26 (31.71%)	1.69 (0.89–3.19)	0.109	1.66 (0.85–3.22)	0.137
Omoro	56 (64.23%)	26 (31.71%)	1.69 (0.89–3.19)	0.109	1.54 (0.79–3.00)	0.206
Level of education						
No formal education	21 (53.85%)	18 (46.15%)	(ref)		(ref)	
Primary education	81 (64.80%)	44 (35.20%)	1.58 (0.76–3.28)	0.221	2.06 (0.93–4.53)	0.073
Secondary education	41 (68.33%)	19 (31.67%)	1.85 (0.80–4.26)	0.148	2.71 (1.10–6.69)	0.030
Tertiary education	15 (68.18%)	7 (31.82%)	1.84 (0.61–5.51)	0.278	2.56 (0.82–8.03)	0.107
Main economic activity						
Livestock farming	33 (67.35%)	16 (32.65%)	(ref)		(ref)	
Crop growing	61 (69.32%)	27 (30.68%)	1.10 (0.51–2.32)	0.812	1.02 (0.48–2.18)	0.957
Business	15 (45.45%)	18 (54.55%)	0.40 (0.16–1.00)	0.051	0.40 (0.16–1.03)	0.058
Both livestock and crop farming	49 (64.47%)	27 (35.53%)	0.88 (0.41–1.88)	0.742	0.79 (0.37–1.69)	0.540

### Bivariate and multivariable analysis of factors influencing practices among farmers

Bivariate analysis was done using simple logistic regression, where *P* < 0.05 was considered statistically significant; the age group 52–85 was significant (cOR: 8.10, 95% CI: 1.07–61.37, *P* = 0.043), with farmers being 8.10 times more likely to have appropriate practices compared with those in the 18–51 years age range. In the multivariable analysis where *P* < 0.05 was considered statistically significant, the age group 52–85 years had a stronger significance (aOR: 11.19, 95% CI: 1.50–83.36, *P* = 0.018) (**Table 5**).

**Table 5:** Bivariate and multivariable analysis of factors associated with appropriate practices.

Variables	Appropriate practices	Inappropriate practices	cOR (95% CI)	*P*-value	aOR (95% CI)	*P*-value
Age						
18–51	167 (86.53)	26 (13.47%)	(ref)		(ref)	
52–85	52 (98.11%)	1 (1.89%)	8.10 (1.07–61.37)	0.043	11.19 (1.50–83.36)	0.018
Gender						
Male	110 (88.71%)	14 (11.29%)	(ref)		(ref)	
Female	109 (89.34%)	13 (10.66%)	1.07 (0.49–2.38)	0.874	1.17 (0.52–2.66)	0.702
District						
Amuru	70 (85.37%)	12 (14.63%)	(ref)		(ref)	
Gulu	73 (89.02%)	9 (10.98%)	1.39 (0.55–3.51)	0.485	1.49 (0.56–3.95)	0.423
Omoro	76 (92.68%)	6 (7.32%)	2.17 (0.77–6.11)	0.142	2.60 (0.88–7.69)	0.085
Level of education						
No formal education	33 (84.62%)	6 (15.38%)	(ref)		(ref)	
Primary education	114 (91.20%)	11 (8.80%)	1.88 (0.65–5.49)	0.246	3.03 (0.97–9.44)	0.056
Secondary education	52 (86.67%)	8 (13.33%)	1.18 (0.38–3.72)	0.775	2.03 (0.58–7.11)	0.275
Tertiary education	20 (90.91%)	2 (9.09%)	1.82 (0.33–9.93)	0.490	3.24 (0.64–16.40)	0.156
Main economic activity						
Livestock farming	45 (91.84%)	4 (8.16%)	(ref)		(ref)	
Crop growing	76 (86.36%)	12 (13.64%)	0.56 (0.17–1.86)	0.345	0.47 (0.13–1.68)	0.243
Business	29 (90.79%)	4 (12.12%)	0.64 (0.15–2.79)	0.557	0.68 (0.15–3.09)	0.618
Both livestock and crop farming	69 (90.79%)	7 (9.21%)	0.88 (0.24–3.17)	0.841	0.83 (0.21–3.27)	0.786

### Association of AMR awareness, antimicrobial use practices, and access to veterinary services across districts, education levels, and training

A chi-square analysis was done where *P* < 0.05 was considered statistically significant, and it showed that there was a significant difference to access to veterinary services across districts (*P* = 0.014). There was a highly significant association between training and appropriate practices in farmers [*X*^2^ (1) = 13.048, *P* = 0.000]. There was no significant association found between education level with correct AMR understanding, following dosage, and seeking veterinary advice (**Table 6**).

**Table 6:** Association of AMR awareness, antimicrobial use practices, and access to veterinary services across districts, education levels, and training.

Variables	Outcome	*X*^2^ (df)	*P*-value
District	Good AMR awareness	3.54 (2)	0.170
	Appropriate AMU	2.25 (2)	0.325
	Access to veterinary services	8.51 (2)	0.014
Education level	Correct AMR understanding	2.69 (3)	0.443
	Follow dosage	2.55 (3)	0.466
	Seeking veterinary advice	3.38 (3)	0.336
Training impact	Good AMR awareness	0.182 (1)	0.670
	Appropriate practices	13.048 (1)	0.000
	Seeking veterinary advice	0.224 (1)	0.636

### Source of information on antimicrobial use and antimicrobial resistance

**Figure 1:**
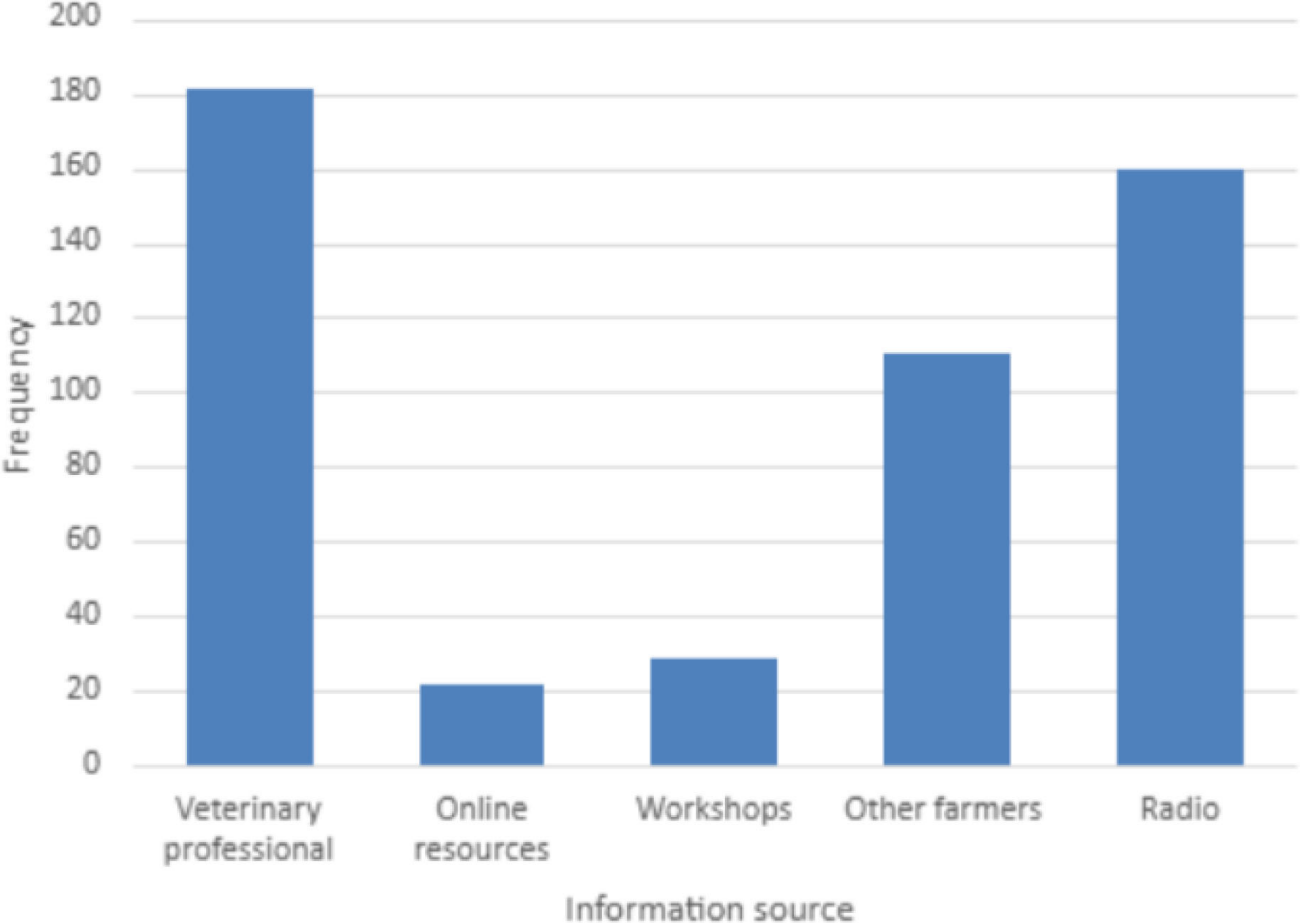
Source of information on antimicrobial use and antimicrobial resistance.

The major source of information was veterinary professionals 181 (73.6%) followed by radio stations (160, 65.0%) (**Figure 1**).

### Challenges in implementing antimicrobial resistance prevention

**Figure 2:**
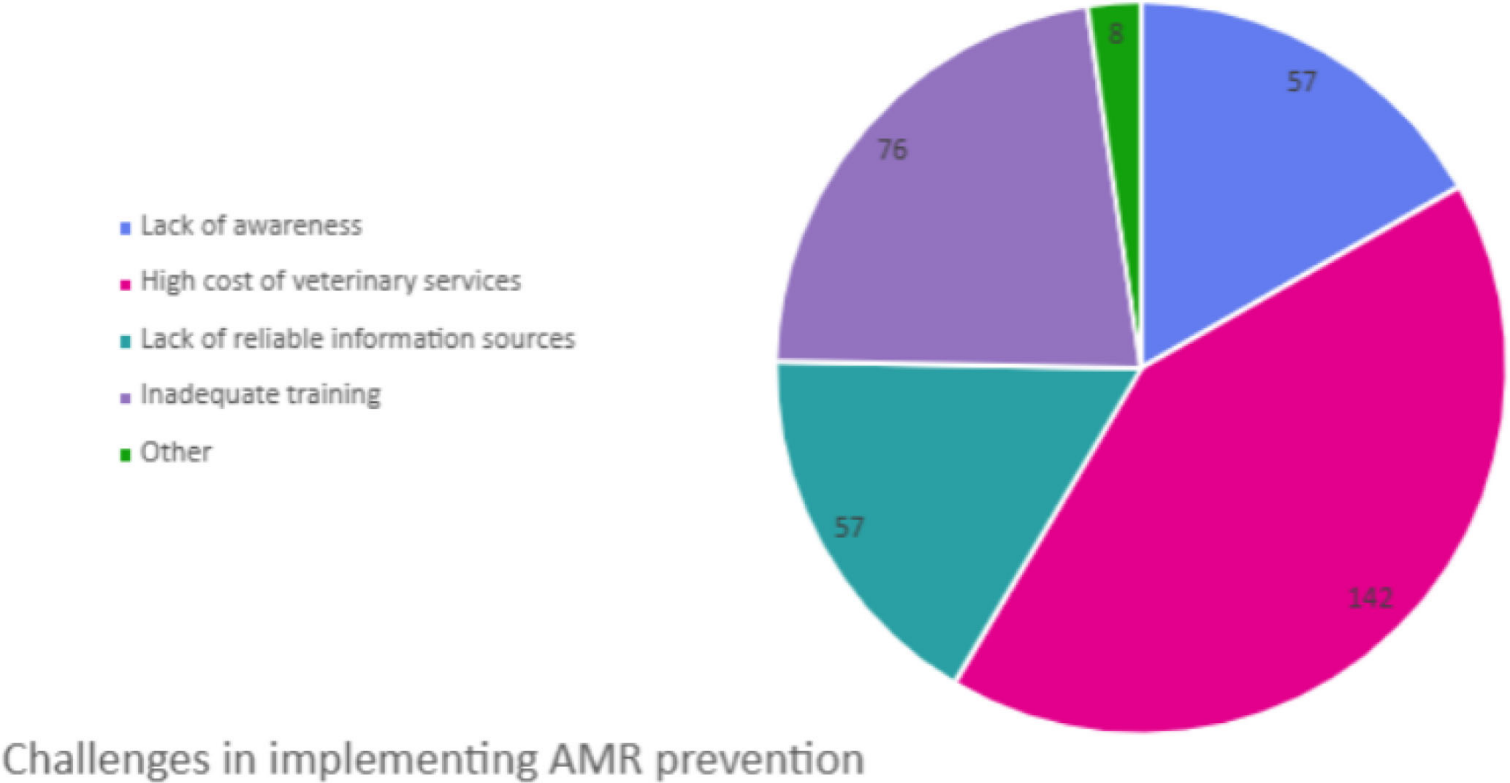
Challenges in implementing antimicrobial resistance prevention.

Most farmers (218, 88.6%) had challenges in implementing antimicrobial resistance prevention practices, which were mostly due to the high cost of veterinary services (142, 65.1%) and inadequate training resources (76, 34.9%) (**Figure 2**).

## Discussion

AMR represents one of the most important human and animal health-threatening issues worldwide (Palma et al., 2020). It has emerged as one of the principal public health problems of the 21st century that threatens the effective prevention and treatment of an ever-increasing range of infection (Prestinaci et al., 2015).

The main economic activity reported showed a larger proportion of farmers who grow crops (35.8%), followed by both livestock and crop farming (30.9%). This could be attributed to the fact that most farmers in Northern Uganda are facing a problem on food security. Thus, they tend to grow more crops for food and sell the excess, and they also find that growing crops is cheaper compared with keeping livestock. Practicing both livestock and crop farming is second most common as this can be linked to some farmers who easily use crop refuse as feeds for animals as well as the droppings of livestock as manure for crop farming. This finding is not surprising as Uganda’s main economic activity is agriculture, including animal husbandry, particularly in rural areas (Bamwesigye et al., 2020).

This study showed a larger median number of 6 to 19 animals reared by farmers compared with a study in Bangladesh where the farmers had six to 10 animals (Hossain et al., 2022). This could be attributed to the presence of more vacant land areas available in northern Uganda plus the cultural pride that comes with having more animals, which makes one be considered richer in the society. However, this implies that the farmers are more likely to use antibiotics, thus a higher chance of AMR occurrence and easy spread.

In this study, approximately 55% of the farmers had heard of antimicrobial resistance. This could be related to the presence of district veterinary professionals who are called upon when animals are sick, for regular vaccination, and sometimes to carry out sensitization talks when administering treatments. Some farmers reported learning AMR from health centers and radio stations. However, in a study done in Mymensingh Division of Bangladesh, a greater proportion of farmers (71.2%) had heard about antimicrobial resistance (Hossain et al., 2022): 85.7% in Kenya (Omolo et al., 2024) and 63.6% in Wakiso District, Uganda (Musoke et al., 2023). This can be related to a lower level of education of the northern region compared with the other regions of the country because of the war which had plagued the region and could have hindered access to education.

Only 35.8% of the farmers in this study who had heard of AMR were reported to have understood its correct meaning in this study compared with a similar study done in Mymensingh Division of Bangladesh where a larger proportion of farmers (56.7%) correctly knew about antimicrobial resistance (Hossain et al., 2022). This could be attributed to the few veterinary professionals who serve a wide area, thus ending up treating the animals in the shortest period and having limited time to educate the farmers. There is also limited funding on programs that teach farmers about AMR, which could be evidenced by a lesser proportion of farmers (32.9%) who reported to have had received training on antimicrobial resistance. This can be linked to financial restraints as most trainings have to be facilitated right from the reading materials to the compensation of the training attendance.

In this study, most participants (35.0%) incorrectly stated that antimicrobials work by preventing disease progression, and AMR is mostly caused by lack of vaccination (36.5%), while in Kenya most participants (23.1%) attributed it to the overuse of antimicrobials (Omolo et al., 2024). This can be linked to most farmers (50.8%) in this study having attained primary education as the highest level, making it hard to correctly understand the mechanism in which the antimicrobials worked. Such topics are also taught to learners at a higher education level in subjects such as agriculture. The analysis using logistic regression also showed that having secondary education was associated with having better awareness compared with having no education. This observation was also reported similarly in a study in Wakiso, Uganda, where most participants (49.3%) attained primary education (Musoke et al., 2023). However, in a study done in Kenya, most participants (31.8%) have completed secondary education (Omolo et al., 2024), which could be linked to having a better understanding.

Seeking veterinary advice was reported by many farmers (54.5%) as a method to prevent antimicrobial resistance. This can be attributed to the fact that majority of the farmers in northern Uganda have access to their district veterinary professionals who are paid by the government. Thus, the farmers must contact them only in case of need of veterinary services. The farmers also know that it is the job of veterinary professionals to take care of the animals when notified. In contrast, 96% of the livestock farmers in Vietnam (Pham-Duc et al., 2019) and 82% of the livestock keepers in Ethiopia reported that immunization could be beneficial in lowering AMR generation (Gemeda et al., 2020).

Most farmers (95.5%) noted antimicrobial resistance as a problem as they reported the consequences of AMR on their farms that would affect the input and output of resources. This can be attributed to the fact that AMR was introduced to the farmers after the first knowledge/awareness questions which may have affected their perfection. This can also be observed as most farmers had good awareness on AMR (158, 64.2%). A similar study done in Wakiso district reported that a lesser proportion of participants (37.1%) mentioned that AMR was only a problem for people who took antimicrobials regularly (Musoke et al., 2023).

Most farmers (75.6%) agreed that antimicrobials should only be used under supervision. The farmers reported in interviews that it is their role to contact the veterinary professionals when the animals are sick and follow the instructions given to them. This ranges from animal checkup, drug prescription, administration, and follow-up, which are similar to a study in Wakiso District where almost all of the participants (96.7%) agreed that people should use antimicrobials only when prescribed by a health professional (Musoke et al., 2023). A previous study in Northeastern Ethiopia reported that more than half of the farmers (54.2%) believed that antibiotics should only be prescribed by veterinarians (Gebeyehu et al., 2021).

Of the farmers interviewed, 82.5% reported antimicrobial use in the last 12 months. Most of the farmers noted using the drugs for routine vaccination of animals and deworming. However, some reported to have used drugs to treat various conditions such as flu, diarrhea, and tick infection. In another study done in Kiambu County, Kenya, 78% reported the use of an antibiotic in the past 6 months prior to the study (Kariuki et al., 2023). Many farmers (64.2%) in this study rarely used antibiotics, which is similar to a study done in Kenya where almost all farmers (92.7%) reported administering antibiotics to their cattle at least once in the last year (Kisoo et al., 2023). The farmers reported to mostly using the drugs occasionally when the animals were sick, which is an appropriate practice.

Most farmers (74.8%) obtain antibiotics and their dosage from veterinary professionals, who also guided their prescription. This is attributed to the availability of veterinary doctors supported by the district local governments who are accessible to local council leaders and the farmers through phones and their offices. This is consistent with the results from a study done in Wakiso District which showed that the farmers obtain antimicrobials for use in animals from veterinary workers (74.5%) and veterinary drug shops (19.1%) (Musoke et al., 2023), which are also similar to the farmers in Lira and Mukono (Nohrborg et al., 2022).

However, in a study done in different African countries, it was revealed that a higher proportion of livestock farmers (83%) get antibiotics from agro-veterinary pharmacies (Caudell et al., 2020), which is similar to 45.5% of Northeastern Ethiopia farmers who get antimicrobials from veterinary pharmacies (Gebeyehu et al., 2021). In contrast, 64% of the animal producers in Turkey took advice from their colleagues and purchase antimicrobials without prescription (Ozturk et al., 2019).

Strength and limitation: The study included a large sample size and addressed a gap in research on Northern Uganda livestock farmers. However, as a limitation, a standard KAP tool was not used, thus future studies should do more studies with it.

## Conclusion

In this study, more than half of the farmers reported having heard of AMR before; however, only 48 (19.5%) understood its correct meaning, which hits a significant knowledge gap. Most of the farmers had good attitude particularly in noting AMR as a problem, with the need to practice responsible antimicrobial use as important and only under supervision. Although majority of the farmers reported seeking veterinary guidance, some farmers have inappropriate practices such as the use of personal experience. These findings suggest an urgent need for education and regulation to prevent the development and spread of AMR in livestock farming. There is a need to organize targeted AMR campaigns to sensitize the community, particularly through veterinary professionals and radio stations which are the major sources of information to the farmers. The government should also introduce strict laws to aid in the AMR fight in livestock.

## Data Availability

The original contributions presented in the study are included in the article/**Supplementary Material**. Further inquiries can be directed to the corresponding author.

## References

[B1] Ayukekbong J. A. Ntemgwa M. Atabe A. N. (2017). The threat of antimicrobial resistance in developing countries: Causes and control strategies. Antimicrob. Resist. Infect. Control 6, 1–8. doi: 10.1186/s13756-017-0208-x. PMID: 28515903 PMC5433038

[B2] Bamwesigye D. Doli A. Adamu K. J. Mansaray S. K. (2020). A review of the political economy of agriculture in Uganda: Women, property rights, and other challenges. Universal J. Agric. Res. 8, 1–10. doi: 10.13189/ujar.2020.080101

[B3] Broens E. M. van Geijlswijk I. M. (2018). Prudent use of antimicrobials in exotic animal medicine. Vet. Clinics North Am.: Exot. Anim. Pract. 21, 341–353. doi: 10.1016/j.cvex.2018.01.014. PMID: 29655474

[B4] Canton L. Lanusse C. Moreno L. (2021). Rational pharmacotherapy in infectious diseases: Issues related to drug residues in edible animal tissues. Animals 11, 2878. doi: 10.3390/ani11102878. PMID: 34679899 PMC8532868

[B5] Caudell M. A. Dorado-Garcia A. Eckford S. Creese C. Byarugaba D. K. Afakye K. . (2020). Towards a bottom-up understanding of antimicrobial use and resistance on the farm: A knowledge, attitudes, and practices survey across livestock systems in five African countries. PloS One 15, e0220274. doi: 10.1371/journal.pone.0220274. PMID: 31978098 PMC6980545

[B6] Gebeyehu D. T. Bekele D. Mulate B. Gugsa G. Tintagu T. (2021). Knowledge, attitude and practice of animal producers towards antimicrobial use and antimicrobial resistance in Oromia zone, north eastern Ethiopia. PloS One 16, 1–15. doi: 10.1371/journal.pone.0251596. PMID: 33979388 PMC8115805

[B7] Gemeda B. A. Amenu K. Magnusson U. Dohoo I. Hallenberg G. S. Alemayehu G. . (2020). Antimicrobial use in extensive smallholder livestock farming systems in Ethiopia: Knowledge, attitudes, and practices of livestock keepers. Front. Vet. Sci. 7. doi: 10.3389/fvets.2020.00055. PMID: 32175334 PMC7055293

[B8] Getahun H. (2023). Antimicrobial resistance Sustainable Development Goals Series (WHO). doi: 10.1007/978-3-031-33851-9_22, PMID:

[B9] Grace D. (2015). Review of evidence on antimicrobial resistance and animal agriculture in developing countries. Available online at: https://cgspace.cgiar.org/server/api/core/bitstreams/d6c7dfdb-4b71-4f54-aabe-874be76fa1be/content (Accessed October 29, 2025).

[B10] Hossain M. T. Rafiq K. Islam M. Z. Chowdhury S. Islam P. Haque Z. . (2022). A survey on knowledge, attitude, and practices of large-animal farmers towards antimicrobial use, resistance, and residues in Mymensingh Division of Bangladesh. Antibiotics 11, 442. doi: 10.3390/antibiotics11040442. PMID: 35453194 PMC9030753

[B11] Ilea R. C. (2009). Intensive livestock farming: Global trends, increased environmental concerns, and ethical solutions. J. Agric. Environ. Ethics. 22, 153–167. doi: 10.1007/s10806-008-9136-3. PMID: 41841152

[B12] Kariuki J. W. Jacobs J. Ngogang M. P. Howland O. (2023). Antibiotic use by poultry farmers in Kiambu County, Kenya: Exploring practices and drivers of potential overuse. Antimicrob. Resist. Infect. Control 12, 1–13. doi: 10.1186/s13756-022-01202-y. PMID: 36604700 PMC9817392

[B13] Kisoo L. Muloi D. M. Oguta W. Ronoh D. Kirwa L. Akoko J. . (2023). Practices and drivers for antibiotic use in cattle production systems in Kenya. One Health 17. doi: 10.1016/j.onehlt.2023.100646. PMID: 38024269 PMC10665206

[B14] Musoke D. Lubega G. B. Obeng M. B. Brandish C. Winter J. Niyongabo F. . (2023). Knowledge, perceptions and practices on antimicrobial resistance in humans and animals in Wakiso district, Uganda: A cross sectional study. PloS Global Public Health 3, 1–16. doi: 10.1371/journal.pgph.0002701. PMID: 38091332 PMC10718425

[B15] Nadeem S. F. Gohar U. F. Tahir S. F. Mukhtar H. Pornpukdeewattana S. Nukthamna P. . (2020). Antimicrobial resistance: More than 70 years of war between humans and bacteria. Crit. Rev. Microbiol. 46, 578–599. doi: 10.1080/1040841X.2020.1813687. PMID: 32954887

[B16] Naghavi M. Vollset S. E. Ikuta K. S. Swetschinski L. R. Gray A. P. Wool E. E. . (2024). Global burden of bacterial antimicrobial resistance 1990–2021: A systematic analysis with forecasts to 2050. Lancet 404, 1199–1226. doi: 10.1016/S0140-6736(24)01867-1. PMID: 39299261 PMC11718157

[B17] Nohrborg S. Dione M. M. Winfred A. C. Okello L. Wieland B. Magnusson U. (2022). Geographic and socioeconomic influence on knowledge and practices related to antimicrobial resistance among smallholder pig farmers in Uganda. Antibiotics 11, 251. doi: 10.3390/antibiotics11020251. PMID: 35203853 PMC8868422

[B18] Omolo J. O. Omani R. Caudell M. A. Kimani T. Kiambi S. Fasina F. O. (2024). Knowledge, attitudes, practices on antimicrobial use in animals among livestock sector stakeholders in Kenya. Vet. Med. Int. 2024, 1–11. doi: 10.1155/2024/8871774. PMID: 39606423 PMC11599476

[B19] Ozturk Y. Celik S. Sahin E. Acik M. N. Cetinkaya B. (2019). Assessment of farmers’ knowledge, attitudes and practices on antibiotics and antimicrobial resistance. Animals 9, 1–13. doi: 10.3390/ani9090653. PMID: 31487911 PMC6770244

[B20] Palma E. Tilocca B. Roncada P. (2020). Antimicrobial resistance in veterinary medicine: An overview. Int. J. Mol. Sci. 21, 1–18. doi: 10.3390/ijms21061914. PMID: 32168903 PMC7139321

[B21] Pham-Duc P. Cook M. A. Cong-Hong H. Nguyen-Thuy H. Padungtod P. Nguyen-Thi H. . (2019). Knowledge, attitudes and practices of livestock and aquaculture producers regarding antimicrobial use and resistance in Vietnam. PloS One 14, 1–15. doi: 10.1371/journal.pone.0223115. PMID: 31553776 PMC6760827

[B22] Prestinaci F. Pezzotti P. Pantosti A. (2015). Antimicrobial resistance: A global multifaceted phenomenon. Pathog. Global Health 109, 309–318. doi: 10.1179/2047773215Y.0000000030. PMID: 26343252 PMC4768623

[B23] Puvača N. (2022). Antimicrobial resistance and treatment in companion, food and exotic animals. Antibiotics 11, 1360. doi: 10.3390/antibiotics11101360. PMID: 36290017 PMC9598238

[B24] Survey A. A. Release S. (2022). “ Annual agricultural survey (AAS) 2019 – statistical release,” in Uganda bureau of statistics, vol. 2019 ( Uganda Bureau of Statistics)

