# Supplementary material for: Awareness and practices regarding antimicrobial resistance among livestock farmers in Northern Uganda
Source: Front Antibiot. 2026 Apr 6;5:1745965. doi: 10.3389/frabi.2026.1745965 (PMC13093969; doi:10.3389/frabi.2026.1745965)
Supplement: Supplementary file 1 [file Table1.docx]

**Results**

**Socio-demographic characteristics of livestock farmers**

Out of the 246 participants interviewed; the median age was 38 years (interquartile range: 29-50). Most farmers had primary education 125 (50.8%) and grew crops as the major economic activity 88 (35.8%). Goats were most reared animal 167 (67.7%) with a median of 10 animals (interquartile range: 6-19). The median distance from nearest drug shop was 2 km (interquartile range: 1-5) and few farmers 15 (6.1%) kept exotic breeds. The median estimated monthly income was 100,000 Ugandan shillings (interquartile range: 30000-200000).

**Table 1: Socio-demographic characteristics of livestock farmers**

| Variable | Sample size | Frequency | Percentage |
| --- | --- | --- | --- |
| **Gender** | 246 |  |  |
| Male |  | 124 | 50.4 |
| Female |  | 122 | 49.6 |
| **Age (median, (interquartile range))** | 246 | 38 | 29-50 |
| District | 246 |  |  |
| Amuru |  | 82 | 33.3 |
| Gulu |  | 82 | 33.3 |
| Omoro |  | 82 | 33.3 |
| **Level of education** | 246 |  |  |
| No formal education |  | 39 | 15.9 |
| Primary education |  | 125 | 50.8 |
| Secondary education |  | 60 | 24.4 |
| Tertiary education |  | 22 | 9.0 |
| **Type of animal** | 246 |  |  |
| Cattle |  | 123 | 50 |
| Pigs |  | 78 | 31.7 |
| Goats |  | 167 | 67.9 |
| Sheep |  | 9 | 3.7 |
| Rabbits |  | 10 | 4.1 |
| Chickens |  | 94 | 38.2 |
| **Number of animals (median, (interquartile range))** | 246 | 10 | 6-19 |
| **Distance from drug shop (median, (interquartile range))** | 246 | 2 | 1-5 |
| **Estimated monthly income (median, (interquartile range))** | 246 | 100,000 | 30,000-200,000 |
| **Main economic activity** | 246 |  |  |
| Livestock keeping |  | 49 | 19.92 |
| Crop growing |  | 88 | 35.8 |
| Business |  | 33 | 13.4 |
| Both livestock and crop farming |  | 76 | 30.9 |
| **Number of farmers who keep exotic breeds** | 246 | 15 | 6.1 |

**Awareness on antimicrobial resistance among Livestock farmers**

More than half of the farmers 134 (54.5%) had heard of AMR, but only 48 (19.5%) understood its correct meaning. Majority 220 (89.4%) noted antimicrobial resistance as important in farming. Most farmers 86 (35.0%) stated antimicrobials work by preventing disease progressing. The main causes of AMR selected were lack of proper vaccination 89 (36.5%) and overuse of antibiotics 88 (36.1%). Most farmers reported increased treatment costs 123 (50.4%) as the main effect of antimicrobial resistance and more than half stated it can be prevented by seeking veterinary advice 133 (54.5%).

**Table 2: Awareness about Antimicrobial resistance among Livestock farmers**

| Variable | Sample size | Frequency(percentage) |
| --- | --- | --- |
| **Heard of antimicrobial resistance** | 246 | 134 (54.5%) |
| **Know the meaning of antimicrobial resistance** | 134 | 48 (19.5%) |
| **How antimicrobials work** | 246 |  |
| Kill the organisms causing the disease |  | 78 (31.7%) |
| Prevent the disease from progressing |  | 86 (35.0%) |
| Boast the immunity of the animal |  | 82 (33.3%) |
| **Importance of antimicrobial resistance in farming** | 246 |  |
| Not importance |  | 26 (10.6%) |
| Important |  | 220 (89.4%) |
| **Causes antimicrobial resistance** | 243 |  |
| Overuse of antibiotics |  | 88 (36.1%) |
| Poor hygiene |  | 85 (34.8%) |
| contaminated feed |  | 73 (29.9%) |
| lack of proper vaccination |  | 89 (36.5%) |
| **Effects of antimicrobial resistance** | 245 |  |
| Reduced animal health |  | 117 (48.0%) |
| Increased treatment costs |  | 123 (50.4%) |
| Decreased productivity |  | 61 (25.0%) |
| Higher risk of disease spread |  | 87 (35.7%) |
| **Prevention of antimicrobial resistance** | 246 |  |
| Improved farm hygiene |  | 85 (34.8%) |
| Rational antibiotic |  | 84 (34.4%) |
| Regular vaccination |  | 91 (37.3%) |
| Avoiding contaminate |  | 58 (23.8%) |
| Seeking veterinary advice |  | 1. 4.5%) |

**Attitude towards antimicrobial resistance among Livestock farmers**

Majority of the farmers noted antimicrobial resistance as a problem 235 (95.5%), responsible antimicrobial use as important 245 (99.6%) and antimicrobial use only under supervision 186 (75.6%). Most of the farmers were willing to attend an antimicrobial training 243 (98.8%) and change their antimicrobial use practices 232 (94.3%).

**Table 3: Attitude towards antimicrobial resistance among Livestock farmers**

| Variable | Sample size | Frequency | Percentage |
| --- | --- | --- | --- |
| Antimicrobial resistance is a problem | 246 | 235 | 95.5 |
| Antimicrobial use only under veterinary supervision | 246 | 186 | 75.6 |
| Willingness to change antimicrobial use practices | 246 | 232 | 94.3 |
| Responsible antimicrobial use is important | 246 | 245 | 99.6 |
| Willingness to attend antimicrobial training | 246 | 243 | 98.8 |

**Practices about antimicrobial resistance among Livestock farmers**

Most farmers 203 (82.5%) used antimicrobials in the last 12 months. Veterinary prescription guiding antimicrobial choice selection 180 (73.2%) where most farmers obtained the drugs and dosage from veterinary doctors 184 (74.8%). The farmers mostly used the antibiotics rarely 158 (64.2) with majority following the recommended dosage 227 (92.3%) and proper withdrawal periods 221 (89.8%). Overall, 81 (32.9%) received training on antimicrobial resistance with more than half getting training from veterinary professions 41 (56.2%).

**Table 4: Practices about antimicrobial resistance among Livestock farmers**

| Variable | Sample size | Frequency | Percentage |
| --- | --- | --- | --- |
| **Use of antimicrobial in last 12 months** | 246 | 203 | 82.5 |
| **Frequency of antibiotics use** | 246 |  |  |
| Weekly |  | 12 | 4.9 |
| Monthly |  | 76 | 30.9 |
| Rarely |  | 158 | 64.2 |
| **Obtaining antibiotics and their dosage** | 246 |  |  |
| Veterinary doctors |  | 184 | 74.8 |
| Drug shops |  | 48 | 19.5 |
| Fellow farmers |  | 2 | 0.8 |
| Personal experience |  | 5 | 2.0 |
| Drug bottle |  | 7 | 2.9 |
| **Following recommended dosage** | 246 | 227 | 92.3 |
| **Following proper withdrawal periods for antibiotics** | 246 | 221 | 89.8 |
| **Training on antimicrobial resistance** | 246 | 81 | 32.9 |
| **Source of training** | 73 |  |  |
| Veterinary professional |  | 41 | 56.2 |
| Private organization |  | 17 | 23.3 |
| Government health organization |  | 4 | 5.5 |
| NGO |  | 11 | 15.0 |
| **Determination of antimicrobial choice** | 246 |  |  |
| Veterinary prescription |  | 180 | 73.2 |
| Personal experience |  | 28 | 11.4 |
| Cost |  | 13 | 5.3 |
| Recommendations from other farmers |  | 25 | 10.2 |

**Source of information on antimicrobial use and antimicrobial resistance**

The major source of information was veterinary professionals 181 (73.6%) followed by radios 160 (65.0%).


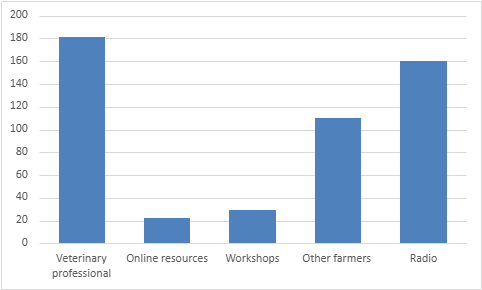


Figure 1: **Source of information on antimicrobial use and antimicrobial resistance**

**Challenges in implementing antimicrobial resistance prevention**

Most farmers 218 (88.6%) had challenges in implementing antimicrobial resistance prevention practices mostly due to the high cost of veterinary services 142 (65.1%) and inadequate training resources 76 (34.9%).

Figure 2: **Challenges in implementing antimicrobial resistance prevention**
